# Characterization of ovarian progenitor cells for their potential to generate steroidogenic theca cells *in vitro*

**DOI:** 10.1530/REP-23-0407

**Published:** 2024-05-31

**Authors:** Xin Wen, Jiexia Wang, Mengjie Qin, Hu Wang, Jingfeng Xu, Xiaoju Guan, Dan Shan, Panpan Chen, Jiajia Xie, Jingjing Shao, Ping Duan, Congde Chen, Haolin Chen

**Affiliations:** 1 Department of Gynecology and Obstetrics, the Second Affiliated Hospital and Yuying Children’s Hospital of Wenzhou Medical University, Wenzhou, Zhejiang, China; 2 Department of Pediatric Urology, Key Laboratory of Children Genitourinary Diseases of Wenzhou City, Key Laboratory of Structural Malformations in Children of Zhejiang Province, the Second Affiliated Hospital and Yuying Children’s Hospital of Wenzhou Medical University, Wenzhou, Zhejiang, China; 3 Department of Pharmacology, the Second Affiliated Hospital and Yuying Children’s Hospital of Wenzhou Medical University, Wenzhou, Zhejiang, China; 4 Zhejiang Provincial Key Laboratory of Anesthesiology, Department of Anesthesiology, the Second Affiliated Hospital and Yuying Children’s Hospital of Wenzhou Medical University, Wenzhou, Zhejiang, China

## Abstract

**In brief:**

Progenitor cells with ovulation-related tissue repair activity were identified with defined markers (LGR5, EPCR, LY6A, and PDGFRA), but their potentials to form steroidogenic cells were not known. This study shows that the cells can generate progenies with different steroidogenic activities.

**Abstract:**

Adult mammalian ovaries contain stem/progenitor cells necessary for folliculogenesis and ovulation-related tissue rupture repair. Theca cells are recruited and developed from progenitors during the folliculogenesis. Theca cell progenitors were not well defined. The aim of current study is to compare the potentials of four ovarian progenitors with defined markers (LY6A, EPCR, LGR5, and PDGFRA) to form steroidogenic theca cells *in vitro*. The location of the progenitors with defined makers was determined by immunohistochemistry and immunofluorescence staining of ovarian sections of adult mice. Different progenitor populations were purified by magnetic-activated cell sorting (MACS) and/or fluorescence-activated cell sorting (FACS) techniques from ovarian cell preparation and were tested for their abilities to generate steroidogenic theca cells *in vitro*. The cells were differentiated with a medium containing LH, ITS, and DHH agonist for 12 days. The results showed that EPCR+ and LGR5+ cells primarily distributed along the ovarian surface epithelium (OSE), while LY6A+ cells distributed in both the OSE and parenchyma. However, PDGFRA+ cells were exclusively located in interstitial compartment. When the progenitors were purified by these markers and differentiated *in vitro*, LY6A+ and PDGFRA+ cells formed steroidogenic cells expressing both CYP11A1 and CYP17A1 and primarily producing androgens, showing characteristics of theca-like cells, while LGR5+ cells generated steroidogenic cells devoid of CYP17A1 expression and androgen production, showing a characteristic of progesterone-producing cells (granulosa- or lutea-like cells). In conclusion, progenitors from both OSE and parenchyma of adult mice are capable of generating steroidogenic cells with different steroidogenic capacities, showing a possible lineage preference.

## Introduction

The ovary has two major functions: generating reproductive cells (eggs) and producing sex hormones, two essential components for successful reproduction. Ovarian hormone disorders are often accompanied by irregular menstruation, amenorrhea, infertility, and other symptoms that affect not only reproduction but also the general health of the individuals (Miller & Auchus 2011). Three major endocrine cells in the ovary are theca cells, granulosa cells, and luteal cells. Theca cells provide androgen precursors for estrogen synthesis by the granulosa cells, while luteal cells produce progesterone to support pregnancy and fetal growth. All three cells are necessary for maintaining healthy sex steroid hormone profile within the ovary and across the body during different reproductive stages (Kotsuji & Tominaga 1994, Dewailly *et al.* 2016). Unlike steroidogenic cells in the testes, ovaries undergo reproductive cycles regularly that involve steroidogenic cell regenerations and transformations. The dynamics of theca steroidogenic cells, especially their progenitor cells, have been not well studied.

The ovarian theca layer surrounds the developing follicles and contains various cell types, including steroidogenic, vascular endothelial, immune, and fibroblast-like cells (Yamamoto *et al.* 1997, Young & McNeilly 2010). Since theca cells are not found in the early follicles and are only observed once a follicle developed with two or more layers of granulosa cells, it suggests that theca cells have to be recruited or newly developed from progenitors during folliculogenesis even in the adult. Isolation and culture of potential steroidogenic theca progenitor cells was carried out for various species, including mouse (Honda *et al.* 2007), porcine (Lee *et al.* 2013), sheep (Adib & Valojerdi 2017), monkey (Chen *et al.* 2021), and human (Dalman *et al.* 2018, Asiabi *et al.* 2020). These studies consistently demonstrated that mammalian ovaries of adults contain theca cell progenitors. However, these *in vitro* studies adopted procedures similar to the culture of general mesenchymal stem cells without pre-purification of the cells. With proper inducing conditions, although these cells can express some theca cell-specific markers and produce androgens, they were also frequently found to express other ovarian cell markers, such as granulosa cells and/or oocytes, suggesting the heterogeneity of the cells in culture.

In addition to these studies focused on steroidogenic theca progenitor cells, researchers have also identified a group of general progenitor cells in both the ovarian surface epithelium (OSE) and interstitial compartments for ovulation-related tissue repair and homeostasis maintenance (Gamwell *et al.* 2012, Ng *et al.* 2014, Schindler *et al.* 2018, Wang *et al.* 2019). During fetal development, progenitors from both the OSE and interstitial perivascular locations contribute cells to granulosa and theca cell populations (Hummitzsch *et al.* 2015, Li *et al.* 2022). The OSE is a single layer of poorly differentiated epithelial cells that contains progenitors to repair the ovarian rupture during ovulation (Ng *et al.* 2014, Schindler *et al.* 2018, Wang *et al.* 2019). Pluripotent stem cells were also reported in the OSE that have the potential to form oocytes recently (Sharma & Bhartiya, 2021). The markers used to identify the progenitor populations included LY6A (Gamwell *et al.* 2012), leucine-rich repeat-containing G protein-coupled receptor 5 (LGR5) (Ng *et al.* 2014, Schindler *et al.* 2018), and endothelial cell protein C receptor (EPCR, coded by gene *Procr*) (Wang *et al.* 2019). However, the relationships between these tissue repair progenitors with defined markers and those steroidogenic progenitors identified by *in vitro* approaches (Honda *et al.* 2007, Lee *et al.* 2013, Adib & Valojerdi 2017, Dalman *et al.* 2018, Asiabi *et al.* 2020) are not clear. Also, it is still unknown whether these ovulation-related tissue repair progenitors with defined markers are capable of forming steroidogenic cells.

In the present study, we compared the ability of the three ovarian progenitors with the distinguished markers (Ly6A, LGR5, and EPCR) to generate steroidogenic theca cells *in vitro.* As a comparison, ovarian cells expressing testicular stem Leydig cell markers platelet-derived growth factor receptor alpha (PDGFRA) and CD51 (Ge *et al.* 2006, Jiang *et al.* 2014, Shao *et al.* 2023) were also tested.

## Materials and methods

### Chemicals and reagents

DMEM/F12 medium, fetal bovine serum (FBS), insulin/transferrin/selenium (ITS), dexamethasone, and BSA were obtained from Sigma-Aldrich. β-Mercaptoethanol, N2 and B27 supplements, and fibroblast growth factor 2 (FGF2) were purchased from Thermo Fisher. Epidermal growth factor (EGF) was from PeproTech (Rocky Hill, NJ, USA). Chicken embryo extract was from USBiological (Salem, MA, USA). Leukemia inhibitory factor (LIF) was from Millipore. Smoothened agonist (SAG) was purchased from Cayman Chemical. Oncostatin M and platelet-derived growth factor BB (PDGFBB) was from ProSpec (East Brunswick, NJ, USA). Human LH was from MyBioSource (San Diego, CA, USA). Anti-R-phycoerythrin (PE) Magnetic Particles and BD IMag Buffer (10×) were from BD Biosciences (Franklin Lakes, NJ, USA). The manufacturers and the dilutions of the antibodies are summarized in Supplementary Table 1 (see section on supplementary materials given at the end of this article). The primers for qPCR can be found in Supplementary Table 2. The information for ELISA kits for progesterone and estradiol assays can be found in Supplementary Table 3.

### Experimental animals

Female C57BL/6 mice were purchased from the Shanghai Laboratory Animal Center (Shanghai, China) at 9 weeks of age. Mice were maintained in the experimental animal center of Wenzhou Medical University under controlled light (14 h light:10 h darkness) and temperature (22°C), with free access to water and mice chow. All animal procedures were performed according to the National Research Council’s Guide for the Care and Use of Laboratory Animals, with the protocol approved by the Wenzhou Medical University Animal Care and Use Committee.

### Immunohistochemistry and immunofluorescence staining of ovarian sections

For immunohistochemistry staining of ovary sections, the tissues were fixed with 4% paraformaldehyde for 24 h, dehydrated with sucrose (20% for 12 h followed by 30% for 12 h), and finally embedded in OCT at −20°C. Frozen sections (8 μm thick) or isolated/cultured cells were washed with PBS three times and then incubated with a mixture of first antibodies (1:100–1:500) overnight at 4°C, followed by a mixture of fluorescent-labeled secondary antibodies (1:1000) at room temperature for 45 min in darkness. Before examination by microscope, a drop of cover-slide sealing solution containing DAPI was used to reveal the cell nucleus. Negative control was performed by replacing primary antibodies with 1% serum of normal (unimmunized) animals (Supplementary Fig. 1). The specificity of some antibodies used (positive control) was confirmed by staining the testis, uterus, or intestine (Supplementary Figs 2 and 3). The studies that used the antibodies previously were also cited in Supplementary Table 1.

For immunohistochemical staining of ovarian tissue, the labeled dextran polymer method was used. The ovarian sections were treated with 3% hydrogen peroxide to quench endogenous peroxidase activity. After treatment with citrate buffer (pH 6) using microwave heating to retrieve antigens, the sections were incubated with the PDGFRA primary antibody at 4°C overnight, followed by the secondary antibody conjugated to horseradish peroxidase (1:3000, Cat# GAR007, MultiSciences Biotech, China) for 1 h at room temperature. The bound antibodies were then visualized using a diaminobenzidine substrate, and the nuclei were counter-stained with hematoxylin.

### Isolation of ovarian progenitor cells

For each ovarian progenitor isolation, 20 randomly cycling adult female mice (40 ovaries) were used. The dissected ovaries were washed with PBS, minced with sterile scissors for 20 s, and digested in 10 mL DMEM/F12 medium containing type IV collagenase (1 mg/mL) at 37°C for 30 min, with slow shaking (90 cycles/min). In total, 300 animals were used for 15 isolations for the entire study. Digested cells were filtered through a 70-μm nylon mesh, washed, and then purified via magnetic/fluorescence-activated cell sorting (MACS/FACS) procedures. The progenitor markers used for cell isolation included LY6A, EPCR, LGR5, PDGFRA, and CD51.

For the isolation of progenitors by MACS procedure, the tagged cells were selected by BD IMag protocol according to the manufacturer’s instructions. The cell pellets were suspended in cold BD IMag (BI) buffer at a density of 1 × 10^7^ cells/mL and stained with PE-conjugated primary antibodies (1:500) at 4°C in darkness for 40 min. After washing and labeling with anti-R-PE magnetic particles (1:100) for 30 min at 4°C, the cells were transferred to a collection tube and immediately placed onto the BD IMag Cell Separation Magnet holder (BD Biosciences) for 8 min. The negative fraction (supernatant) was collected, while the positive fraction (adhered to the wall of the tube) was resuspended with BI buffer. The sorting procedure was repeated two to three times depending on the antibodies used. The final positive and negative cell fractions were combined, and the percentage of positive cells was determined by flow cytometer or cultured *in vitro* for differentiation. Some of the positive and negative fractions were collected and had their expressions of steroidogenic genes/proteins (Cyp11a1 and Cyp17a1) checked by qPCR and/or immunofluorescence staining.

### Culture and differentiation of the progenitor cells

The cells isolated by different makers were expanded and differentiated *in vitro* using a protocol well established for differentiating testicular stem Leydig cells (Li *et al.* 2016, Shao *et al.* 2023). In brief, cells were expanded in DMEM/F12 medium containing FBS (5%), ITS (1×), penicillin–streptomycin (100 IU/mL–100 µg/mL), dexamethasone (0.5 nM), LIF (0.5 ng/mL), chicken embryo extract (2.5%), β-mercaptoethanol (50 μM), non-essential amino acids (0.5%), FGF2 (10 ng/mL), EGF (10 ng/mL), PDGFBB (10 ng/mL), oncostatin M (10 ng/mL), and N2 (0.5%) and B27 (1%) supplements. The medium was changed every other day until cells reached approximately 60% confluence. To examine the cell proliferating activity, cells were cultured for 3 days and then labeled with ethynyl-2′-deoxyuridine (EdU, 2 μM) for 24 h. Incorporated EdU was revealed by Click-iT reaction, and the positive cells were counted and expressed as a percentage of total cells as previously reported (Wang *et al.* 2019, Shao *et al.* 2023).

After cells reached about 60% confluence, they were transferred to a differentiation-inducing medium consisting of DMEM/F12, 1× ITS, 0.5 μM SAG, and 2 ng/mL LH and were differentiated for up to 12 days. The medium was changed every other day, and the spent medium was saved for steroid hormone assays by UPLC-MS/MS or ELISA kits (Shao *et al.* 2023). RNA was isolated from the cells, and the expressions of Cyp11a1, Cyp17a1, Lhcgr, and Fshr were assayed by qPCR. The steroidogenic proteins, including CYP11A1, CYP17A1, and HSD3B, were revealed immunofluorescently or enzymatically.

### Immunofluorescent and enzymatic staining of marker proteins

Cells isolated or cultured were washed with PBS three times and then incubated with a mixture of first antibodies (1:100–1:500, see Supplementary Table 1) overnight at 4°C, followed by a mixture of fluorescent-labeled secondary antibodies (1:1000) at room temperature for 45 min in darkness. Before examining by microscope, a drop of cover-slide sealing solution containing DAPI was used to reveal the cell nucleus.

For HSD3B enzymatic staining, freshly isolated cells or cultured cells were allowed to dry on slides for about 10 min. The cells were then stained for 40 min with a solution containing 5β-androstan-3β-ol-17-one steroid substrate (0.4 mM), NAD (1 mg/mL), and tetranitro blue tetrazolium (0.2 mg/mL). After staining, cells were washed with HBSS and fixed with 10% formalin/HBSS for 5 min.

### Steroid hormone assays

Culture medium was collected from four individual experiments. Androstenedione and testosterone were measured by XEVO TQD triple quadrupole mass spectrometer (MS) (Waters Corp, MA, USA) following Acquity UPLC separation as previously reported (Shao *et al.* 2023). Androgen-free cell culture media was used for internal control. The androgen-d3 stock solutions were used to create the internal standard (IS) working solution. The collected cell medium (50 µL) and 100 µL acetonitrile were combined with the IS working solution (5 µL). After vibration for 3 min, the mixture was centrifuged at 12,000 ***g*** for 15 min. The supernatant (10 µL) was loaded into the system through a self-sampling protocol.

Progesterone and estradiol were assayed by ELISA kits (Elabscience Biotech, Shanghai, China). Fifty microliters of culture medium were used for the assay. For experimental accuracy, all samples are assayed in duplicates, with an intra-assay coefficient of variation less than 5%. The detailed information about the progesterone and estradiol ELISA kits can be found in Supplementary Table 3.

### RNA extraction and real-time qPCR analysis

After cell sorting or immediately after culture, total RNA was extracted by RNeasy Mini Kit (Qiagen) according to the manufacturer’s instructions. Extracted RNA was quantified by NanoDrop 2000 Spectrophotometer (Thermo Fisher) and reverse-transcripted to cDNA using the iScript cDNA synthesis kit (Bio-Rad). The qPCR amplification was carried out with a SYBR Green PCR Master Mix Kit, using a protocol consisting of 95°C for 5 min, followed by 40 cycles of 95°C (10 s), and 60°C (30 s). The total 15 μL reaction volume contained 7.5 μL SYBR Green mix, 1.5 μL forward and reverse primer mix, and 0.02 μg diluted cDNA. A universal expression gene ribosomal protein S16 (Rps16) was used as an internal control. The expression levels were calculated using the Delta-Ct method and adjusted to Rps16. The primer sequences can be found in Supplementary Table 2.

### Statistical analysis

Student *t*-test was applied to detect a significant differences for experiments involving two groups. One-way analysis of variance (ANOVA) was used for multiple group comparisons. Differences between groups were considered significant if *P* < 0.05. The significant differences among individual groups were determined by the Student–Newman–Keuls test, using SPSS (IBM) statistical software package. Significances with different confidence levels were defined at *P* < 0.05, 0.01, 0.001, or 0.0001.

## Results

### Distribution of progenitor cells in adult mouse ovaries

**Figure 1:**
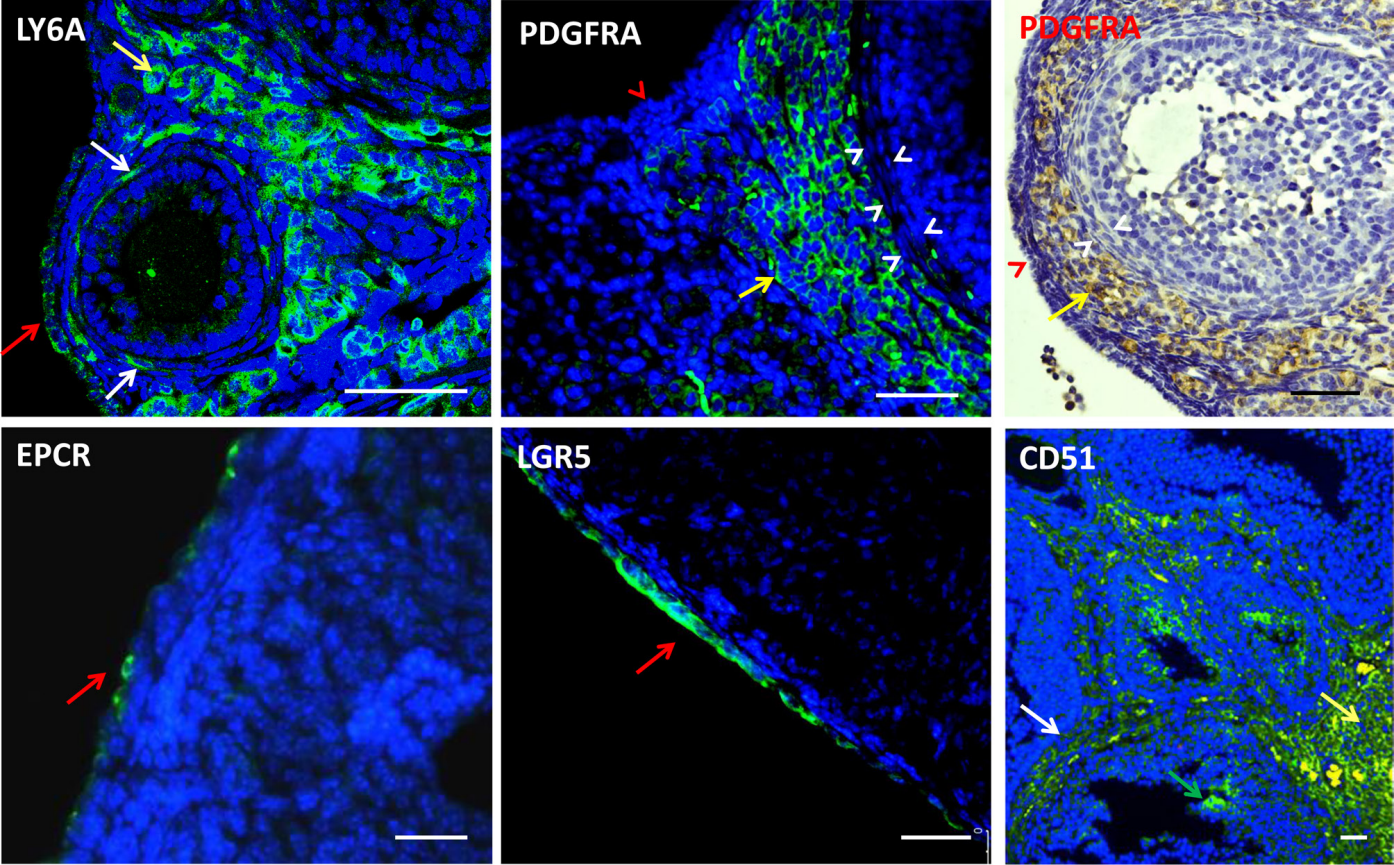
Immunofluorescent staining of five potential stem cell markers. LY6A and PDGFRA primarily labeled ovarian interstitial cells without any staining of granulosa cells, while EPCR and LGR5 primarily labeled OSE cells. LY6A also labeled OSE cells. CD51, however, stained almost all of the interstitial cells and also a few granulosa cells in the follicles. Red arrow: positive OSE cells; red arrowhead: negative OSE cells; white arrowhead: positive theca cells; white arrowhead: negative theca cells; yellow arrow: positive interstitial cells; and green arrow: positive granulosa cells. Scale bars represent 50 μm in length.

Before establishing the differentiating potential of various ovarian progenitors, we first determined their locations in the adult mouse ovary. Ovarian sections were stained with antibody against the three progenitor cell markers, LGR5 (Ng *et al.* 2014, Schindler *et al.* 2018), EPCR (Wang *et al.* 2019), or LY6A (Gamwell *et al.* 2012). For comparison, we also included two well-established testicular stem Leydig cell markers, PDGFRA (Ge *et al.* 2006) and CD51 (Jiang *et al.* 2014, Shao *et al.* 2023) (Fig. 1). As reported previously, LY6A, EPCR, and LGR5 were all expressed by cells primarily associated with the OSE (red arrow), with very few (EPCR) or none (LGR5) positive cells being detected in the interstitial compartment. However, significant numbers of LY6A+ cells were found in the interstitial compartment (yellow arrow), including a few in the theca layer (white arrow). PDGFRA+ cells were exclusively detected in the interstitial compartment (yellow arrow) without any in the OSE (red arrowhead) or theca layer (white arrowhead). CD51, on the other hand, labeled almost all cell types across the whole interstitial compartment, including a few inside follicles (green arrow). When the primary antibodies were replaced by 1% unimmunized animal sera, no staining was detected (Supplementary Fig. 1), confirming the specificities of the staining.

### Isolation and expansion of progenitor cells *in vitro*

**Figure 2:**
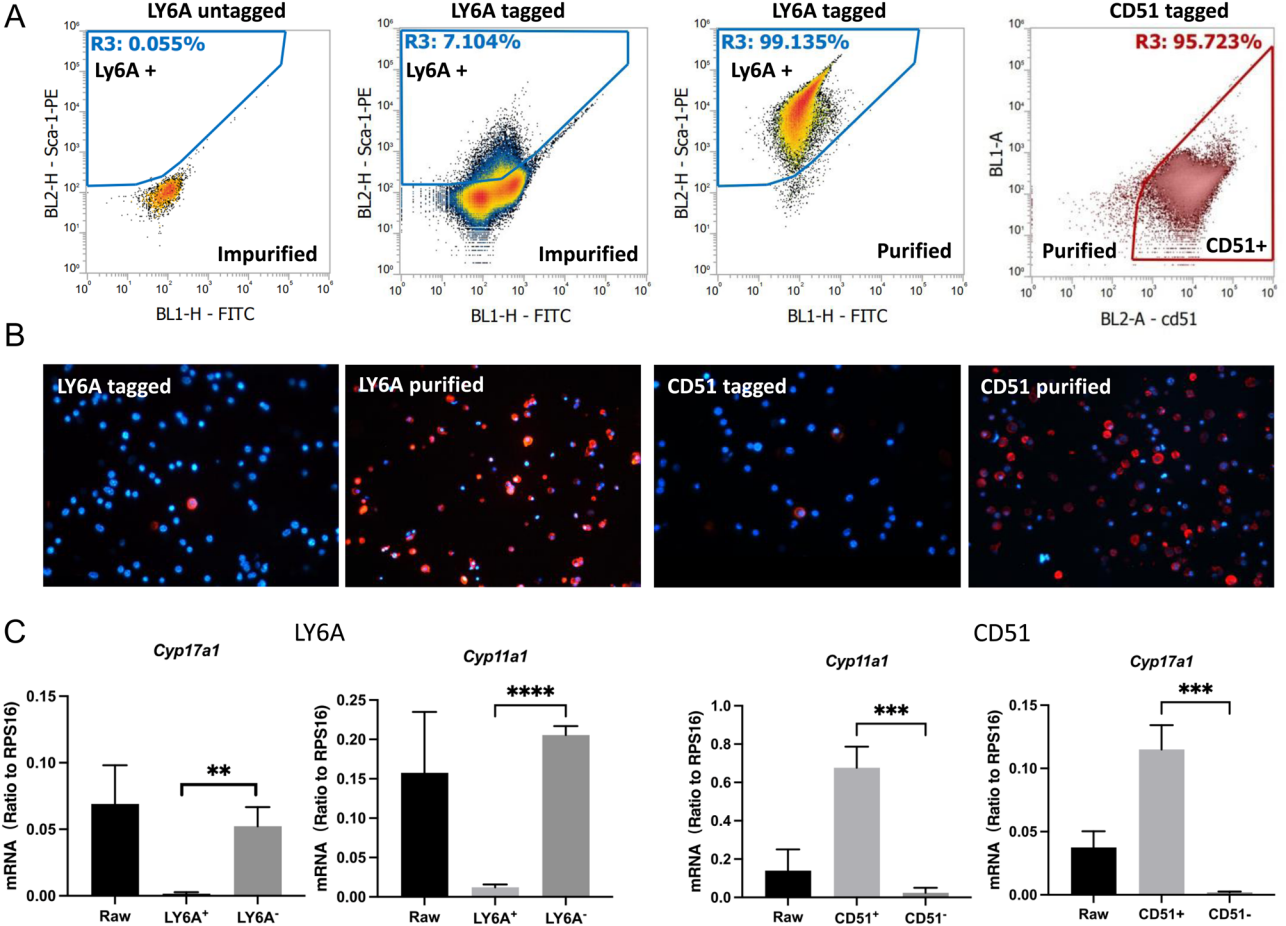
Purification of LY6A and CD51 positive cells by FACS/MACS procedures. (A) Ovarian cells with LY6A-PE antibody staining were isolated by MACS and analyzed by flow cytometer. LY6A+ cells were purified to above 99%, while CD51+ cells were purified to above 95%. Positive cells were within the blue (LY6A) or red (CD51) boxes. LY6A untagged: cells before LY6A PE-antibody staining; LY6A-tagged: cells after LY6A PE-antibody staining but before isolation; LY6A+: LY6A+ cells isolated by MACS procedure; CD51+: CD51+ cells isolated by MACS procedure. (B) PE-tagged LY6A+ and CD51+ cells before and after purification. (C) qPCR analysis of the two steroidogenic genes for ovarian cells before and after enrichments by LY6A or CD51. Data are expressed as mean ± SE of three to four individual experiments. *, **, ***, ****: significantly different from the positive cells at *P* < 0.05, 0.01, 0.001, and 0.0001, respectively.

To compare the potential of these progenitors in generating steroidogenic cells, we isolated the positive cells specifically expressing each marker. The fluorescence-labeled or magnetic particle-tagged cells were purified through MACS and/or FACS procedures (Fig. 2, Supplementary Figs 4, 5, and 6). Taking LY6A as an example, the raw cell suspension before antibody labeling formed a cluster below the diagonal line (Fig. 2A and Supplementary Figure 4; LY6A untagged). For cells labeled with PE-LY6A antibody, about 7.1% were tagged and moved above the diagonal line (Fig. 2A and B, LY6A-tagged). After the FACS procedure or four rounds of MACS purifications, the cell purity reached above 99% (Fig. 2A and B and Supplementary Fig. 4; LY6A purified). Similarly, CD51+, PDGFRA+, EPCR+, and LGR5+ cells were all enriched significantly to about 70–96% (Fig. 2A and B, Supplementary Figs 5 and 6).

To further examine whether the cell preparations isolated by each marker were contaminated with steroidogenic cells, the unsorted, positively and negatively sorted cells were compared for their expressions of the key steroidogenic genes Cyp11a1 (a marker for all ovarian steroidogenic cells) and Cyp17a1 (a marker for theca steroidogenic cells only) (Fig. 2C). Both Cyp11a1 and Cyp17a1 mRNA levels were significantly enriched in LY6A− preparation, and the levels became undetectable in the LY6A+ fraction, suggesting successful exclusion of all steroidogenic cells by LY6A marker (Fig. 2C). Similar enrichments were also achieved for LGR5+ and PDGFRA+ cells. Interestingly, the expressions of the two marker genes were also enriched by CD51, but in exactly the opposite direction to those of LY6A (Fig. 2C). The CD51 positive, but not negative, fraction was enriched for steroidogenic cells. Similarly, for an unknown reason, EPCR also failed to exclude either of the two steroidogenic genes examined, damping the usefulness of CD51 and EPCR in isolating progenitors without contamination of steroidogenic cells.

**Figure 3:**
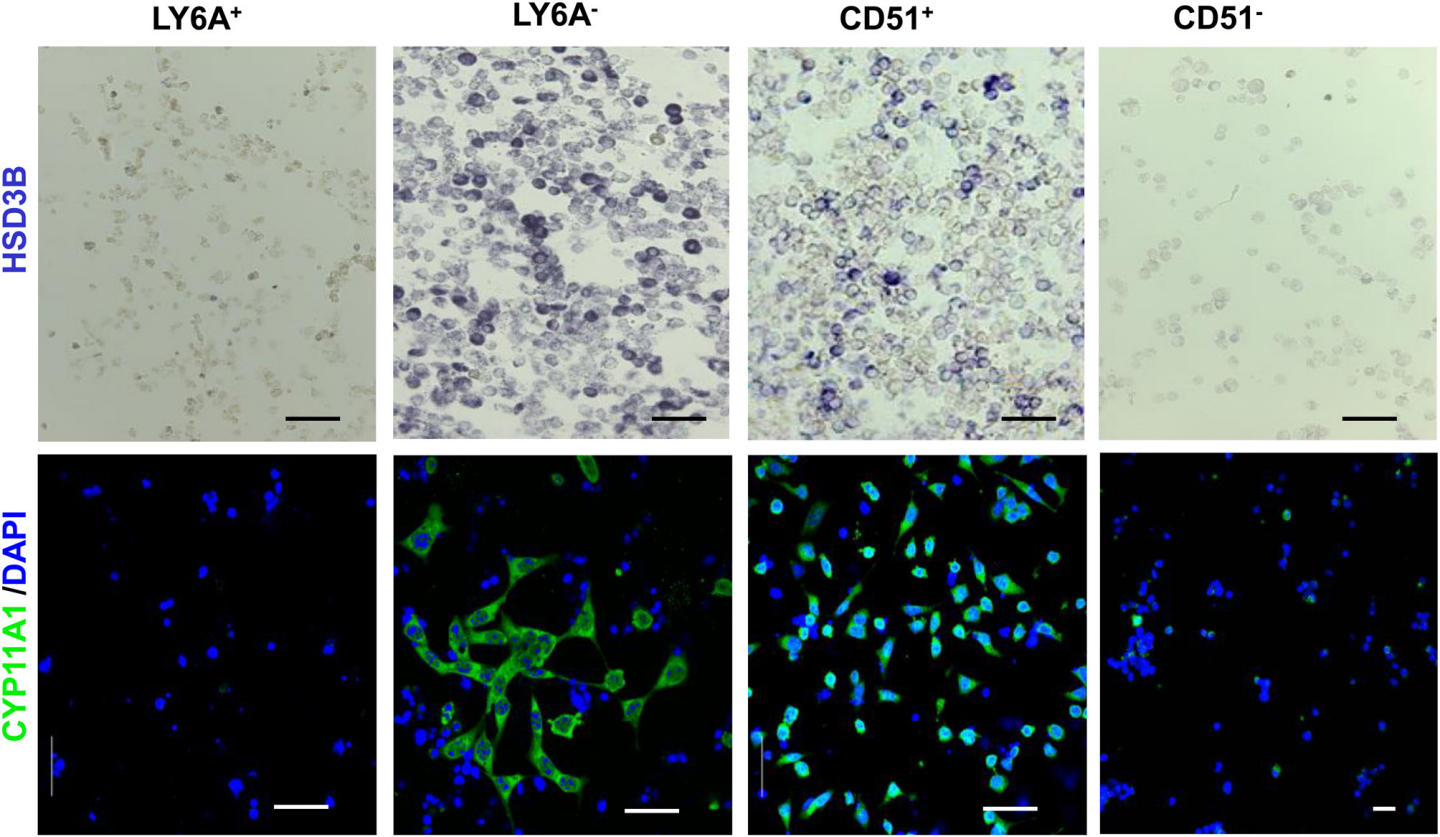
Expression of steroidogenic markers (HSD3B and CYP11A1) by the cells isolated with LY6A and CD51 markers. LY6A− and CD51+ cells exclusively expressed the two steroidogenic proteins, while LY6A+ and CD51− cells did not express the two proteins. Scale bars represent 50 μm in length.

Since LY6A and CD51 behaved in an opposing manner, differences between the cells isolated by the two markers were further compared for their expressions of steroidogenic enzymes HSD3B and CYP11A1 (Fig. 3). Consistent with the qPCR results, LY6A−, but not LY6A+ cells, expressed both HSD3B and CYP11A1, while the opposite was true for CD51 cells.

### Differentiation of progenitor cells into steroidogenic cells *in vitro*

**Figure 4:**
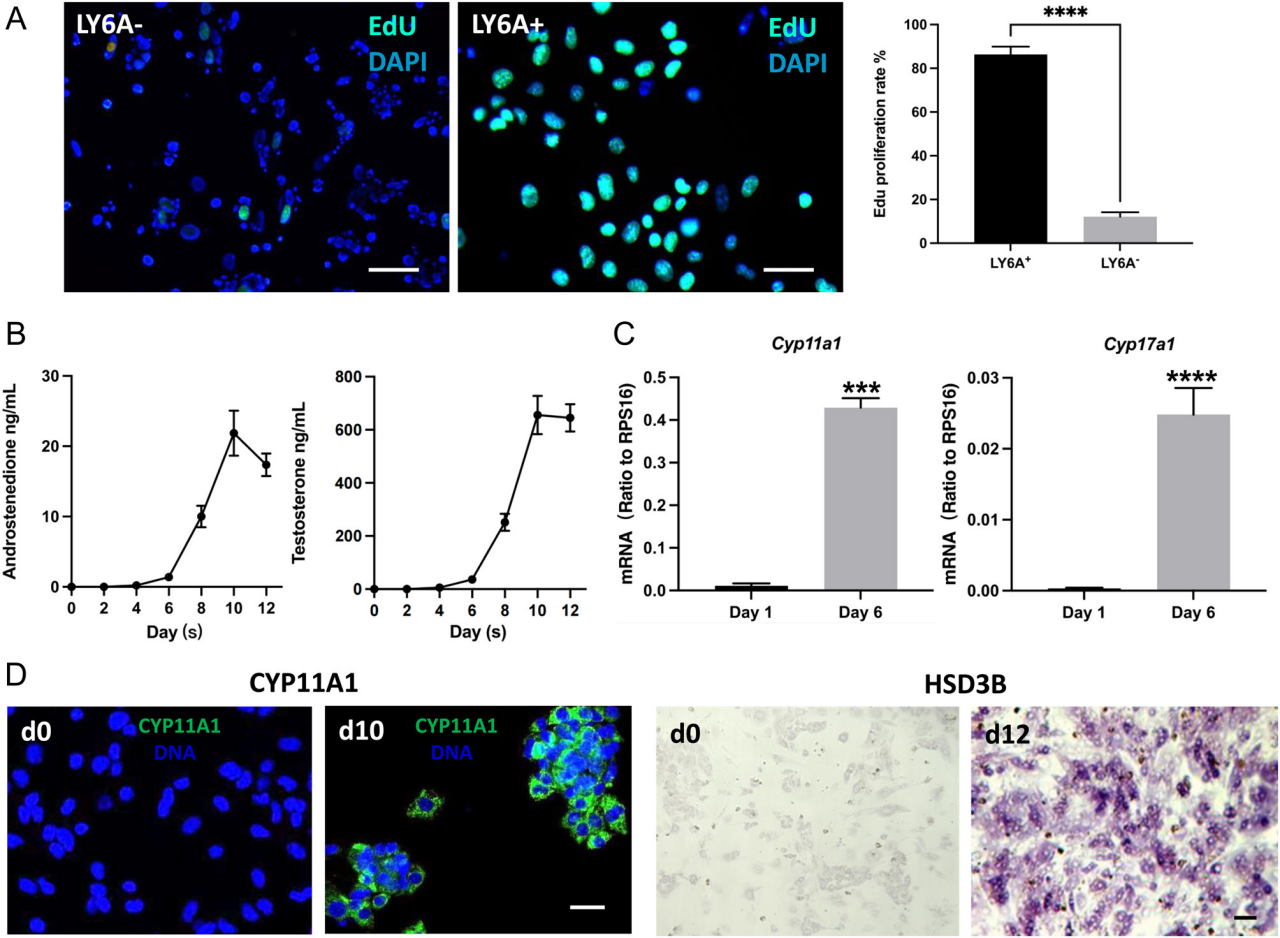
Proliferation and differentiation of LY6A−/+ cells *in vitro*. (A) EdU labeling of LY6A−/+ cells in culture. (B) Androgen (androstenedione and testosterone) production by LY6A+ cells after their steroidogenic cell-inducing differentiation for 12 days. (C) Expression of two steroidogenic genes by LY6A+ cells before and after their differentiation *in vitro* for 6 days. (D) CYP11A1 immunofluorescent and HSD3B enzymatic staining of LY6A+ cells before (d0) and after (d10 or d12) their differentiations *in vitro* for up to 12 days.

To further characterize the differentiating potential of LY6A+ cells, we cultured both LY6A+ and LY6A− cells *in vitro* and assessed their abilities to proliferate and to differentiate into steroidogenic cells. The proliferative activity was assayed by incubating cells with EdU for 24 h during the expanding period. About 87% of LY6A+ cells were labeled with EdU, while only 14% of LY6A− cells were positive for EdU (Fig. 4A).

To determine the potential of LY6A+ cells to form steroidogenic theca cells, a protocol for differentiating testicular stem Leydig cells was adopted (Li *et al.* 2016, Shao *et al.* 2023), with a differentiation-inducing medium containing LH (2 ng/mL), SAG (a DHH agonist, 0.25 μM), and 1× ITS. During the first 4 days of differentiation, no testosterone or androstenedione was detected in the culture medium (Fig. 4B). However, both androgens became detectable by day 6, and the concentrations kept increasing through day 10. To confirm the theca cell identity, expressions of the key steroidogenic genes (*Cyp11a1* and *Cyp17a1*) were assayed for the cells before and after their differentiation (Fig. 4C). No expression was detected for the two genes before the differentiation, but significant levels were detected after the differentiation. Also, the cells were negative for CYP11A1 and HSD3B before the differentiation (d0, Fig. 4D) and became positive for the two enzymes after differentiation for 10 or 12 days (d10 or d12, Fig. 4D).

### Difference in the progenies generated by progenitors *in vitro*

**Figure 5:**
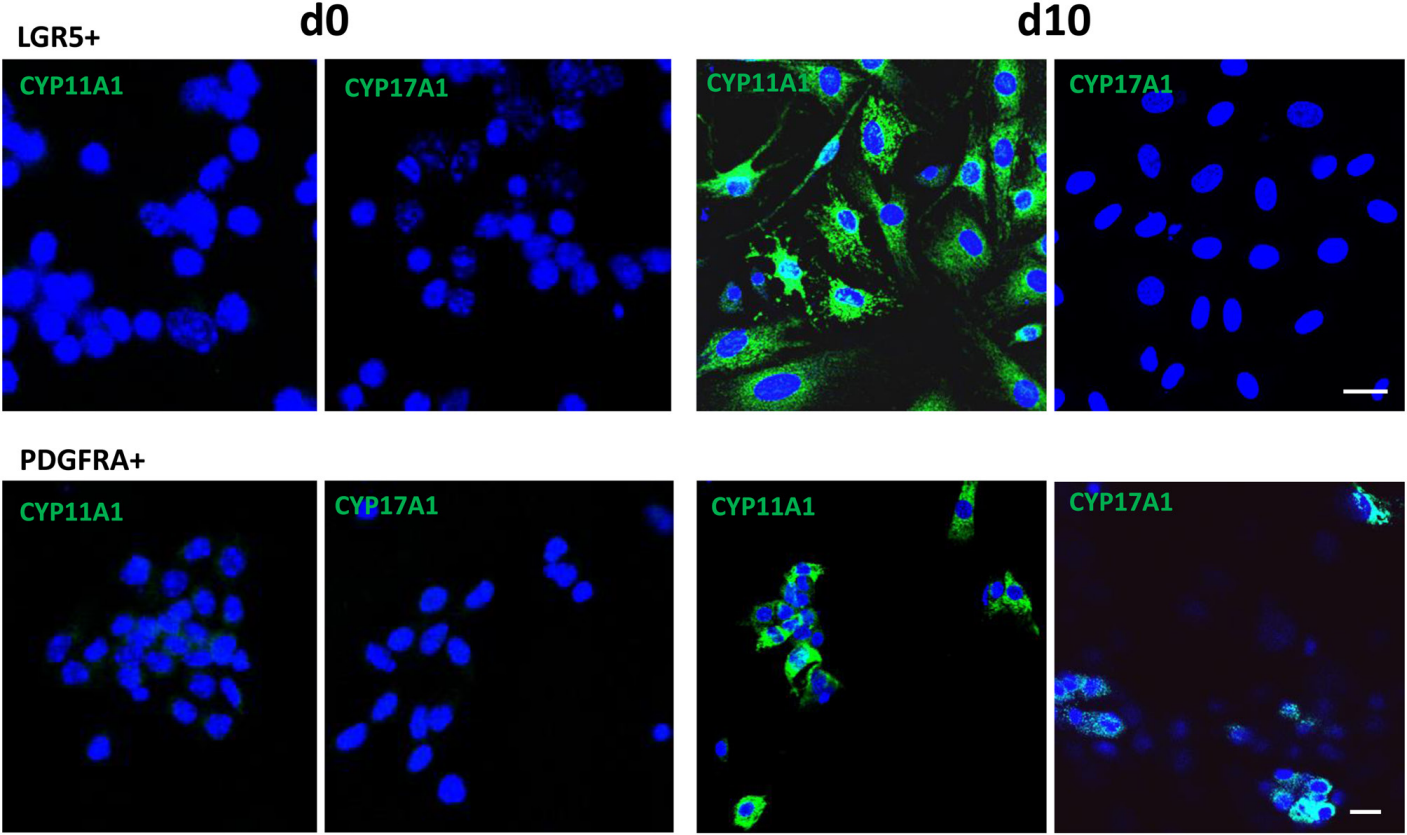
Differentiation of LGR5+ and PDGFRA+ ovarian progenitor cells by steroidogenic cell-inducing medium *in vitro* for 10 days. Expressions of CYP11A1 and CYP17A1 were compared between the two cell types before (d0) and after differentiation for 10 days (d10). Both cells did not express any of the two steroidogenic proteins before the differentiation but expressed CYP11A1 after differentiation (d10). However, LGR5+ cells did not express CYP17A1 while PDGFRA+ cells did after differentiation for 10 days. Scale bars represent 10 μm in length.

Since LY6A+ cells were distributed in both the OSE and parenchyma, the LY6A+ cells that gave rise to steroidogenic cells could come from one or both locations. To further confirm the differences between the progenitors of the two locations, cells isolated by LGR5 (exclusively OSE-associated) and PDGFRA (exclusively parenchyma-associated) were compared for their abilities to generate steroidogenic cells (Fig. 5). The isolated LGR5+ cells and PDFRA+ cells did not express either CYP11A1 or CYP17A1 (Fig. 5, d0). After differentiation *in vitro* for 10 days, the majority of LGR5+ cells gained the ability to express CYP11A1 (Fig. 5, d10), while none of the cells expressed CYP17A1. However, after similar differentiation *in vitro* for 10 days, PDGFRA+ cells were able to generate cells expressing both CYP11A1 and CYP17A1 (Fig. 5, d10), suggesting their theca-like steroidogenic cell characteristics.

**Figure 6:**
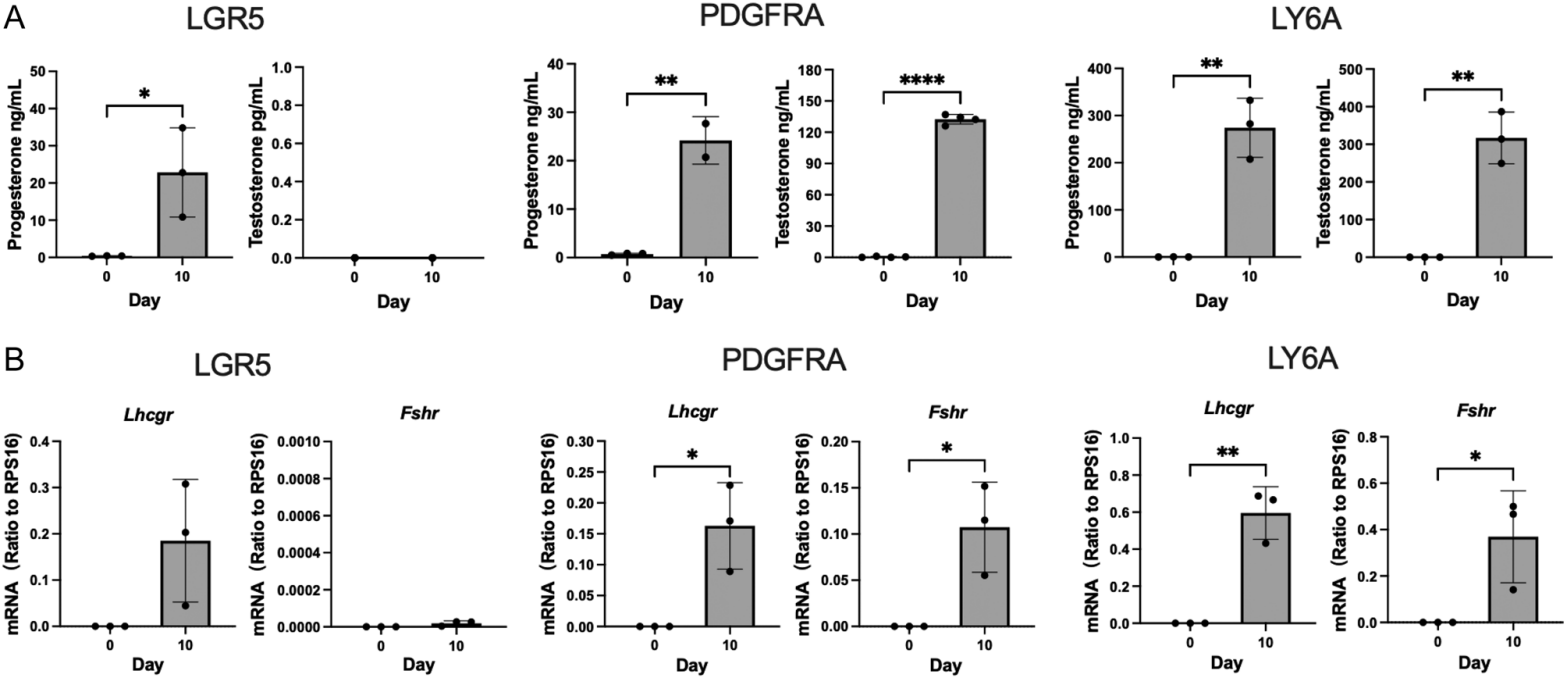
Steroid hormone production and gonadotropin receptor gene expressions. (A) Progesterone and testosterone production by steroidogenic cells generated from LGR5, PDGFRA, or LY6A progenitors. (B) Lhcgr and Fshr expressions by steroidogenic cells generated from LGR5, PDGFRA, or LY6A progenitors. Data are expressed as mean ± SE of three to four individual experiments. *, **, ****: significantly different from the day 10 differentiated cells at *P* < 0.05, 0.01, and 0.0001, respectively.

To further confirm the functional differences of the cells generated by different progenitors, steroid hormones were assayed (Fig. 6A). Cells generated from LGR5+ progenitors produced only progesterone but not testosterone, while cells generated by LY6A+ or PDGFRA+ progenitors were able to produce both progesterone and testosterone. Estradiol was also assayed but was undetectable in all the cells tested. To examine whether there was a difference in the ability of cells to respond to pituitary hormones, *Lhcgr* and *Fshr* were assayed (Fig. 6B). Unexpectedly, cells derived from LGR5+ progenitors expressed *Lhcgr* but not *Fshr*, while cells from LY6A+ or PDGFRA+ progenitors expressed both genes (Fig. 6B).

## Discussion

Ovary somatic progenitor cells have been studied over the years for their abilities to generate functional theca steroidogenic cells. Putative theca stem cells have been reported from various species based on their abilities to give rise to steroidogenic theca-like cells *in vitro* (Honda *et al.* 2007, Lee *et al.* 2013, Adib & Valojerdi 2017, Dalman *et al.* 2018, Asiabi *et al.* 2020, Chen *et al.* 2021). However, except for the few studies that used the theca layer to isolate cells, the true identities and locations of these cells in other studies are difficult to determine since the studies cultured cells without pre-purification. In addition to these studies focused on theca precursors, progenitors involving ovulation-related tissue rupture repairs were also identified along the OSE or interstitial compartment with defined markers (LGR5, EPCA, LY6A, and PDGFRA) (Gamwell *et al.* 2012, Ng *et al.* 2014, Schindler *et al.* 2018, Wang *et al.* 2019). However, the abilities of these progenitors with defined markers to form steroidogenic cells were not known. In the present study, we tested a hypothesis that in addition to their tissue-repairing activities, these progenitors with defined markers may also play roles in forming steroidogenic theca cells. The results confirmed our hypothesis that the progenitors expressing LGR5, LY6A, or PDGFRA were all able to form steroidogenic cells *in vitro*, suggesting their potential roles in contributing steroidogenic cells to adult ovaries.

During every ovulation, the OSE and the underlying tunica albuginea are damaged. The progenitor cells in the remaining OSE start to proliferate and repair the damaged surface (Flesken-Nikitin *et al.* 2013). Various markers (LGR5, EPCA, and LY6A) were identified for OSE progenitors involved in ovulatory rupture repairs (Gamwell *et al.* 2012, Ng *et al.* 2014, Rastetter *et al.* 2014, Schindler *et al.* 2018, Wang *et al.* 2019). LGR5+ cells were found to be broadly expressed during ovary organogenesis but limited to the OSE in neonatal life (Ng *et al.* 2014, Schindler *et al.* 2018). In adults, the expression of Lgr5 is predominantly restricted to proliferative regions of the OSE. Using *in vivo* lineage tracing tools, the Lgr5+ population was demonstrated to play important roles in OSE homeostasis maintenance and ovulatory regenerative repairs (Ng *et al.* 2014, Schindler *et al.* 2018). In the present study, we not only confirmed the specific OSE distributions of the LGR5+ population but also demonstrated their activities to form progesterone-producing cells, suggesting their potential contribution to progesterone-producing cells in the adult ovary.

In addition to LGR5, EPCA is another protein identified recently that can specifically label OSE cells with ovulatory rupture repair activity (Wang *et al.* 2019). EPCA and LGR5 may not mark the same population along the OSE because EPCA cells had distinct lineage-tracing behavior in OSE homeostasis maintenance (Wang *et al.* 2019). In the current study, we also confirmed that the EPCA population is distributed along the OSE. Since the antibody used for cell isolation was not able to exclude the steroidogenic cells from the EPCA+ population, the differential activity of progenitors was not tested in the current study.

LY6A is a glycosylphosphatidylinositol-linked cell surface protein known to be capable of enriching somatic stem cells in other tissues (Spangrude *et al.* 1988, Welm *et al.* 2002, Xin *et al.* 2005). The LY6A+ population was also isolated from the mouse ovary and expanded *in vitro* (Gamwell *et al.* 2012). The cells showed stem/progenitor properties with extensive cell proliferation potential and sphere formation capacity. Unlike LGR5 or EPCA, LY6A only labeled a very rare OSE population, since the LY6A+ cells only represented 2% of the total OSE cells (Gamwell *et al.* 2012). The current study confirmed that the LY6A+ cells were distributed primarily in the interstitial compartment instead of the OSE. PDGFRA, however, is a receptor expressed by mesenchymal cells to respond to PDGF signaling from the epithelium compartment in many tissues. The protein was found useful in labeling and isolating stem Leydig cells from the testes (Ge *et al.* 2006). In the current study, we confirmed its interstitial location in the adult ovary and further demonstrated that the positive cells can be differentiated into theca-like cells *in vitro*.

Since LY6A+ cells are distributed in both the OSE and parenchyma, the isolated progenitors based on the marker could originate from one or both locations. To further study the potential difference in the cells between the two locations, we compared LGR5+ OSE progenitors and PDGFRA+ parenchymal progenitors for their potential to form different steroidogenic cells *in vitro*. The results showed that while both LGR5+ and PDGFRA+ progenitors formed steroidogenic cells, the progenies generated were different. Although the cells generated by progenitors from both locations expressed CYP11A1 and produced progesterone, only cells derived from PDGFRA+ progenitors expressed CYP17A1 and produced androgens, indicating that OSE progenitors may not be able to generate theca-like cells as interstitial PDGFRA+ progenitors do. These results suggest a potential lineage preference between the cells from the two locations. This is consistent with early observations that during fetal development, progenitors from the interstitial perivascular location can contribute cells to both granulosa and theca populations (Hummitzsch *et al.* 2015), while progenitors associated with the OSE only generate granulosa cells (Rastetter *et al.* 2014, Li *et al.* 2022). This preference apparently is maintained up to adulthood.

Interestingly, there is an important difference between the theca-like cells generated in the current study and the real theca cells *in vivo*. Theca cells express LHCGR and respond to LH, but not to FSH (Sharma & Bhartiya, 2021). However, the theca-like cells generated in the present study expressed both Lhcgr and Fshr. The reason for this inconsistency is unknown but may well be related to the simple inducing medium used. The differentiation medium used in the current study was based on previous observations that LH and DHH signaling were enough to induce stem Leydig cells to differentiate (Chen *et al.* 2019, Shao *et al.* 2023). It is well known that theca and Leydig cells share many characteristics, including origins, major products produced (androgens and INSL3), and responses to LH but not to FSH. More importantly, their development depends on a common hedgehog signaling (Yao *et al.* 2002, Wijgerde *et al.* 2005, Spicer *et al.* 2009, Liu *et al.* 2015). The present results indicated that a combination of LH and DHH was also effective in inducing ovarian progenitors into theca-like cells. The differentiated cells of interstitial origin produced androgens but not estrogen, confirming their theca cell identity. However, the unexpected expression of Fshr suggests that the simple formula may not be good enough to induce ‘perfect’ theca cells *in vitro*. Improvement of the medium would be needed in the future.

Isolation and culture of theca progenitor cells was done in different species before (Honda *et al.* 2007, Lee *et al.* 2013, Adib & Valojerdi 2017, Dalman *et al.* 2018, Asiabi *et al.* 2020, Chen *et al.* 2021). Cells cultured by these studies were not pre-purified, so the *in vivo* identity of the cells was not always clear. Characterization of the cells, after they were stably established *in vitro*, showed that the cells shared general mesenchymal stem cell properties most of the times. Differentiations to theca lineage were carried out by complex formulas containing three to nine growth factors in addition to ITS, LH, and/or FSH. The growth factors used included IGF1, SCF, BMP6, TGFB, HGF, KGF, GDF9, EGF, FGF2, LIF, GNDF, and PDGF. Although androgen-producing cells were differentiated, estradiol was also detected in the culture media or was not tested at all, so the true theca cell identity was not always proved. Since these growth factors were shown to play roles in theca cell development, they become the best candidates to be tested with the present differentiation medium. Similarly, the cells established by these procedures also deserve to be tested by the inducing medium established in the present study.

In summary, we have examined the location of ovarian progenitors using four previously reported markers and also have determined their potential to form steroidogenic progenies *in vitro*. LGR5-marked progenitors were exclusively located along the OSE, while PDGFRA-labeled progenitors were found to be located in the interstitial compartment. LY6A-labeled cells, on the other hand, were found in both locations. *In vitro*, LGR5+ OSE progenitors formed CYP11A1+ steroidogenic cells devoid of CYP17A1 expression and testosterone production, while PDGFRA+ interstitial progenitors generated steroidogenic cells expressing both CYP11A1 and CYP17A1 and produced androgens, suggesting a lineage preference of the progenitors from the two locations in forming steroidogenic progenies (progesterone- vs androgen-producing cells). Future studies are needed to confirm such activities* in vivo*.

## Supplementary Materials

Supplementary Material

## Declaration of interest

The authors declare that the study was conducted in the absence of any commercial or financial relationships that could be construed as a potential conflict of interest.

## Funding

This work was supported by grants from the Wenzhou Major Scientific and Technological Innovation Project, CN (ZY2019002), Natural Science Foundation of Zhejiang Provincehttp://dx.doi.org/10.13039/501100004731, CN (Z23H040003 and Q23H040007), and the National Natural Science Foundation of Chinahttp://dx.doi.org/10.13039/501100001809, CN (91949123, 82071626). These sources did not influence the study design; the collection, analysis, or interpretation of data; or the writing of the paper.

## Author contributions

JS and JW: conceptualization, investigation, methodology, formal analysis, data curation, visualization, and writing. XW, JX, FH, XG, and XH: methodology, data curation, and validation. PD, HC, and CC: conceptualization, supervision, project administration, funding acquisition, and writing. All authors contributed to the article and approved the submitted version of the manuscript.
