# Supplementary material for: Characterization of ovarian progenitor cells for their potential to generate steroidogenic theca cells in vitro
Source: Reproduction. 2024 May 31;168(1):e230407. doi: 10.1530/REP-23-0407 (PMC11227037; doi:10.1530/REP-23-0407)
Supplement: Supplementary Material [file supplementary_material.pdf]

Supplementary Materials

Table S1: Antibody for immunofluorescence

Antibody	Host	Vendor	Cat No.	Dilution
**EPCR[1]**	Goat	R&D	AF2749	1:100
**LGR5**	Rabbit	absin	Abs133978	1:500
**LY6A**	Rat	Novus	NBP2-11871	1:200
**CD51**	Rat	BD Pharmingen	611012	1:200
**PDGFRA[2]**	Rabbit	CST	3174T	1:100
**CYP11A1**	Rabbit	Novus	NBP2-92879	1:200
**CYP17A1[3]**	Rabbit	CST	94004S	1:100
**Nestin[4]**	Rabbit	Novus	NB100-1604	1:200
**Alexa-Fluor488-conjugated Donkey-Anti-Rabbit IgG(H+L)**	**Donkey**	Yeasen	34206ES	1:200
**Alexa-Fluor488-conjugated Donkey-Anti-Rat IgG(H+L)**	**Donkey**	Yeasen	34406ES	1:200
**Alexa Fluor488-conjugated Donkey-Anti-Goat IgG(H+L)**	**Donkey**	Yeasen	34306ES	1:200

Antibody for flow cytometry

Antibody	Host	Vendor	Cat No.	Dilution
**LY6A[5]**	Rat	Invitrogen	12-5981-82	1:100
**CD51[6]**	Rat	Invitrogen	12-0512-82	1:100
**EPCR[7]**	Rat	Invitrogen	12-2012-82	1:100
**LGR5[8]**	Rat	R&D	FAB8240P	1:100
**PDGFRA[9]**	Rat	Invitrogen	12-1401-81	1:100

References that used the antibodies previously:

[1] Toyama, H, Arai F, Hosokawa K, et al. N-cadherin+ HSCs in fetal liver exhibit higher long-term bone marrow reconstitution activity than N-cadherin- HSCs [J]. Biochem Biophys Res Commun, 2012, 428(3): 354-359. DOI:10.1016/j.bbrc.2012.10.058.

[2] Zhou, T, Li Y, Li X, et al. Microglial debris is cleared by astrocytes via C4b-facilitated phagocytosis and degraded via RUBICON-dependent noncanonical autophagy in mice [J]. Nat Commun, 2022, 13(1): 6233. DOI:10.1038/s41467-022-33932-3.

[3] Xia, K, Wang F, Lai X, et al. AAV-mediated gene therapy produces fertile offspring in the Lhcgr-deficient mouse model of Leydig cell failure [J]. Cell Rep Med, 2022, 3(11): 100792. DOI:10.1016/j.xcrm.2022.100792.

[4] Hsu, T W, Lu Y J, Lin Y J, et al. Transplantation of 3D MSC/HUVEC spheroids with neuroprotective and proangiogenic potentials ameliorates ischemic stroke brain injury [J]. Biomaterials, 2021, 272: 120765. DOI:10.1016/j.biomaterials.2021.120765.

[5] Li, N, Wang Y, Wang A, et al. STS1 and STS2 Phosphatase Inhibitor Baicalein Enhances the Expansion of Hematopoietic and Progenitor Stem Cells and Alleviates 5-Fluorouracil-Induced Myelosuppression [J]. Int J Mol Sci, 2023, 24(3). DOI:10.3390/ijms24032987.

[6] Yokota, T, McCourt J, Ma F, et al. Type V Collagen in Scar Tissue Regulates the Size of Scar after Heart Injury [J]. Cell, 2020, 182(3): 545-562.e523. DOI:10.1016/j.cell.2020.06.030.

[7] Wang, D, Wang J, Bai L, et al. Long-Term Expansion of Pancreatic Islet Organoids from Resident Procr(+) Progenitors [J]. Cell, 2020, 180(6): 1198-1211.e1119. DOI:10.1016/j.cell.2020.02.048.

[8] Kumar, B, Adebayo A K, Prasad M, et al. Tumor collection/processing under physioxia uncovers highly relevant signaling networks and drug sensitivity [J]. Sci Adv, 2022, 8(2): eabh3375.DOI:10.1126/sciadv.abh3375.

[9] Chao, C C, Gutiérrez-Vázquez C, Rothhammer V, et al. Metabolic Control of Astrocyte Pathogenic Activity via cPLA2-MAVS [J]. Cell, 2019, 179(7): 1483-1498.e1422. DOI:10.1016/j.cell.2019.11.016.

[10] Zang ZJ, Wang J, Chen Z, Zhang Y, Gao Y, Su Z, Tuo Y, Liao Y, Zhang M, Yuan Q, Deng C, Jiang MH, Xiang AP. Transplantation of CD51(+) Stem Leydig Cells: A New Strategy for the Treatment of Testosterone Deficiency. Stem Cells. 2017, 35(5):1222-1232. doi: 10.1002/stem.2569.

[11] Ishikawa K, Sugimoto S, Oda M, Fujii M, Takahashi S, Ohta Y, Takano A, Ishimaru K, Matano M, Yoshida K, Hanyu H, Toshimitsu K, Sawada K, Shimokawa M, Saito M, Kawasaki K, Ishii R, Taniguchi K, Imamura T, Kanai T, Sato T. Identification of Quiescent LGR5(+) Stem Cells in the Human Colon. Gastroenterology. 2022 Nov;163(5):1391-1406.e24. doi: 10.1053/j.gastro.2022.07.081.

[12] Kondreddy V, Keshava S, Esmon CT, Pendurthi UR, Rao LVM. A critical role of endothelial cell protein C receptor in the intestinal homeostasis in experimental colitis. Sci Rep. 2020 Nov 25;10(1):20569. doi: 10.1038/s41598-020-77502-3.

Table S2. Primer sequences used for Real-time quantitative PCR

Target gene	Forward primer(5'-3')	Reverse primer(5'-3')
Cyp11a1	GAAGTCTGGAGGCAGGTTGAG	ACCTATTCCGCTTTTCCTTTG
Cyp17a1	ATCTTGGCTTGTATCAGAATG	ACTTGGAAATGATAAAGGAAC
Lhcgr	GACGACGCTAATCTCGCTGG	GAGTAGGATGACGTGGCGAT
Fshr	GAATCCCTGTTCCTCGGCTC	CCCTGACCTATCTGCCATGC
Rps16	TTTGAGATGGACTGTCGGATC	AAGTTACTGGAGCCTGTTTTG

Table S3. Specifications of ELISA kits.

	Company	Catalog	Detection Range	Intra-assay coefficient of variability (%)	Inter-assay coefficient of variability (%)
Progesterone	Elabscience Biotechnology	E-OSEL-M006	78.13-5000pg/mL	3.15	4.98
Estradiol	Elabscience Biotechnology	E-OSEL-M0008	3.13-200 pg/mL	4.65	4.94

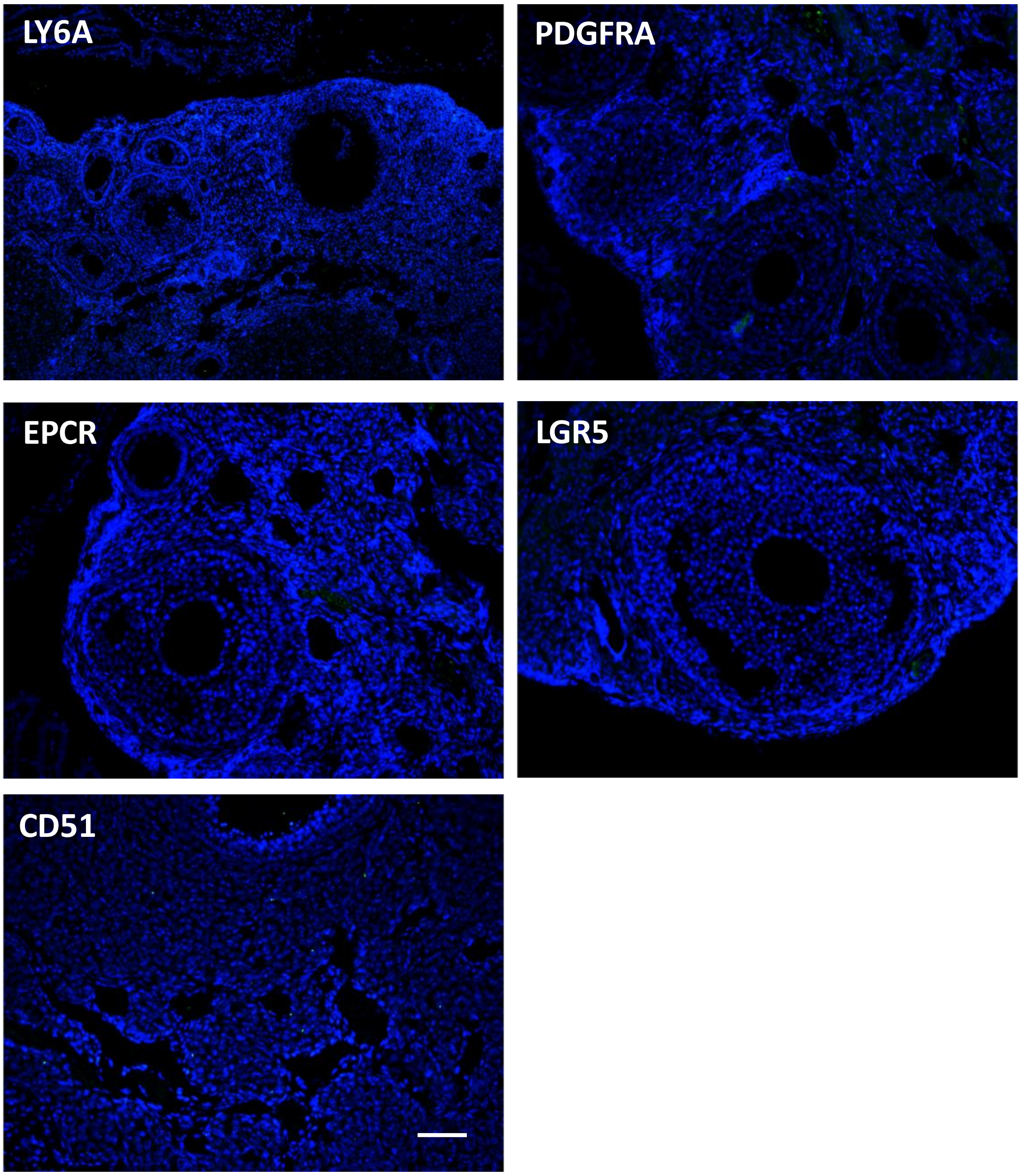

Supplementary Figure 1: Negative control of the five antibodies used for immunofluorescence staining of ovary sections. The first antibodies were replaced with 1% serum of normal (unimmunized) animals during the staining process.
Scale bar: 100μm.

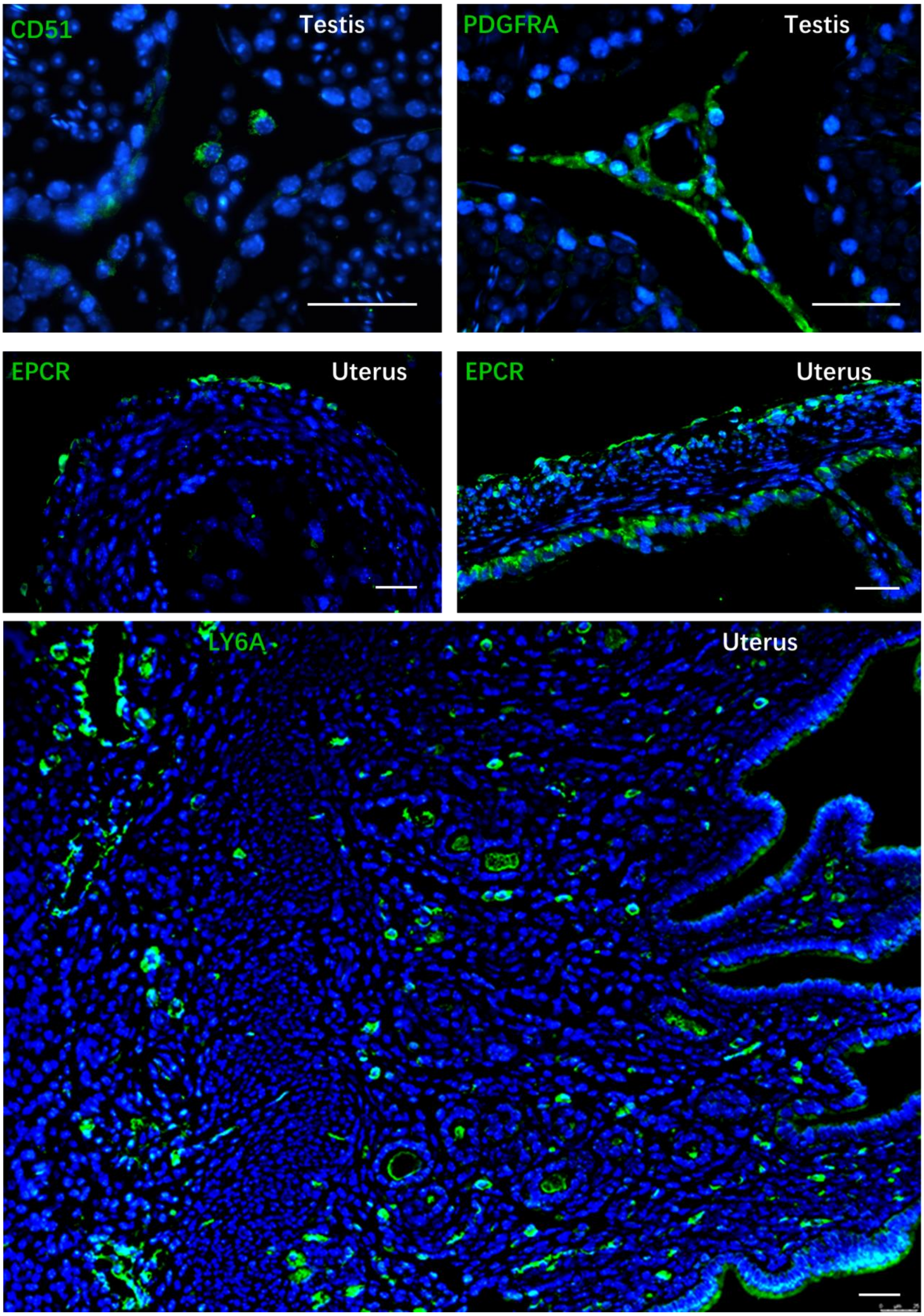

Supplementary Figure 2: Positive control of the four antibodies used for immunofluorescence staining of testis and uterus sections. CD51+ and PDGFRA+ cells are in interstitial compartment of testis[10]; EPCR+ cells are in both uterus surface epithelial and endothelial layers; LY6A+ cells are in uterus endothelium, vascular (sinusoidal) endothelium and as individual cells in stromal area. Scale bar: 50μm.

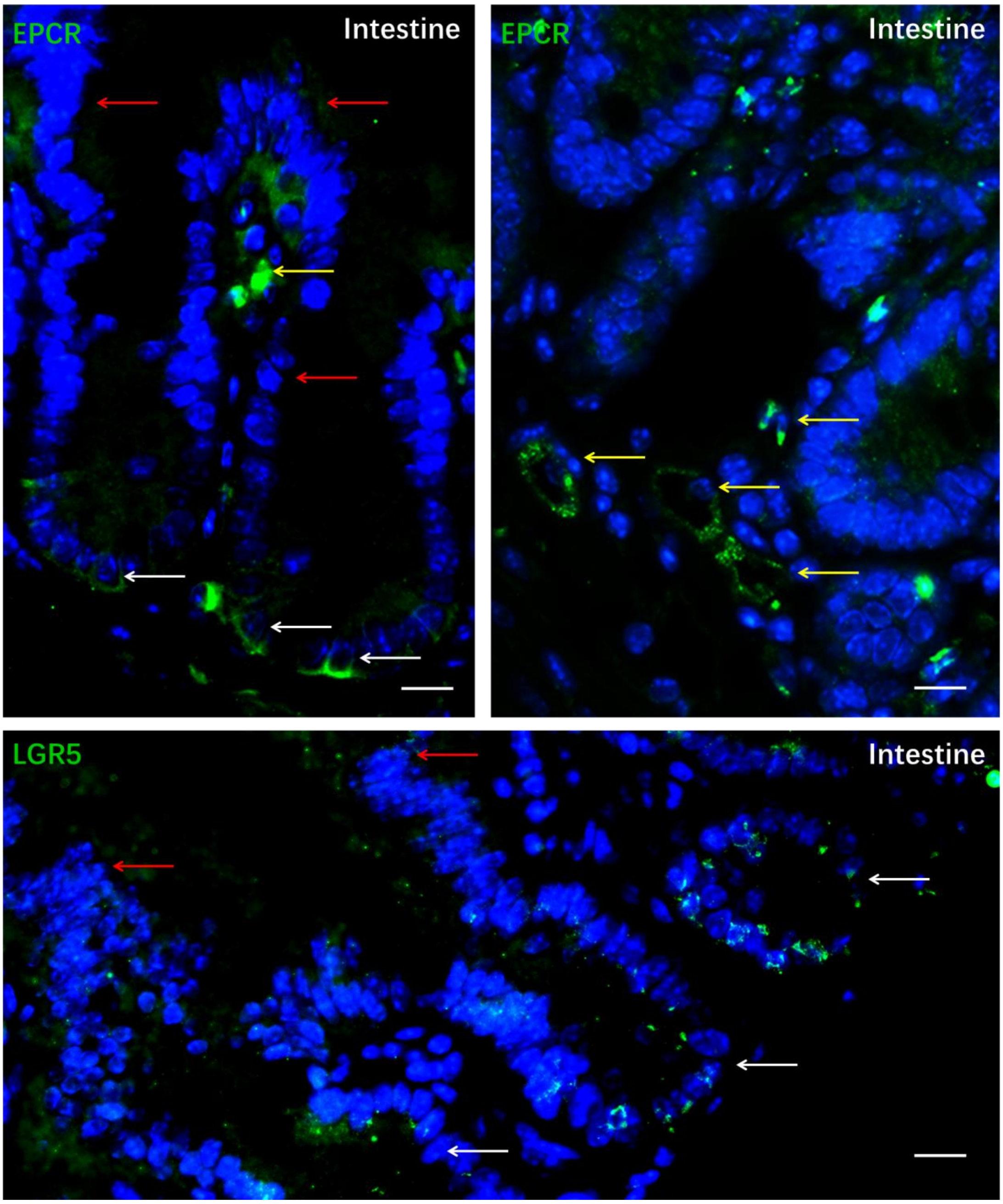

Supplementary Figure 3: immunofluorescence staining of intestine sections by EPCR and LGR5 antibodies. White arrow: positive epithelial cells in crypt base. Red arrows: negative epithelial cells in the middle and tip ranges of crypt. Yellow arrows: EPCR was also found on the endothelium lining blood vessels of the mucosa and in cells surrounding the crypts. These expression patterns are consistent with the ones reported previously [11,12]. Scale bar: 25μm.

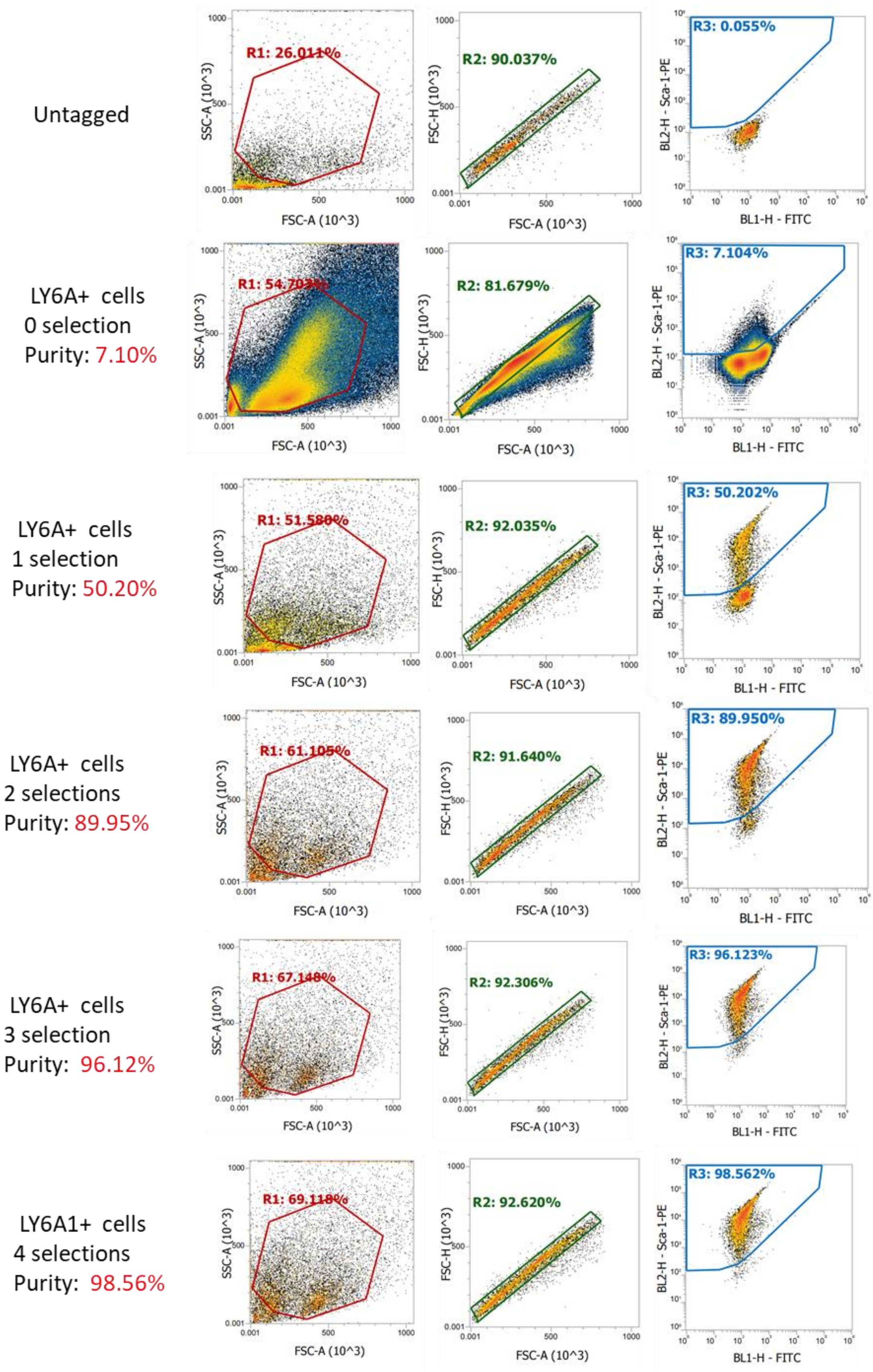

Supplementary Figure 4: Relationship between the purity of LY6A+ cells and the repeats of magnetic Assisted Cell Sorting (MACS) selections.

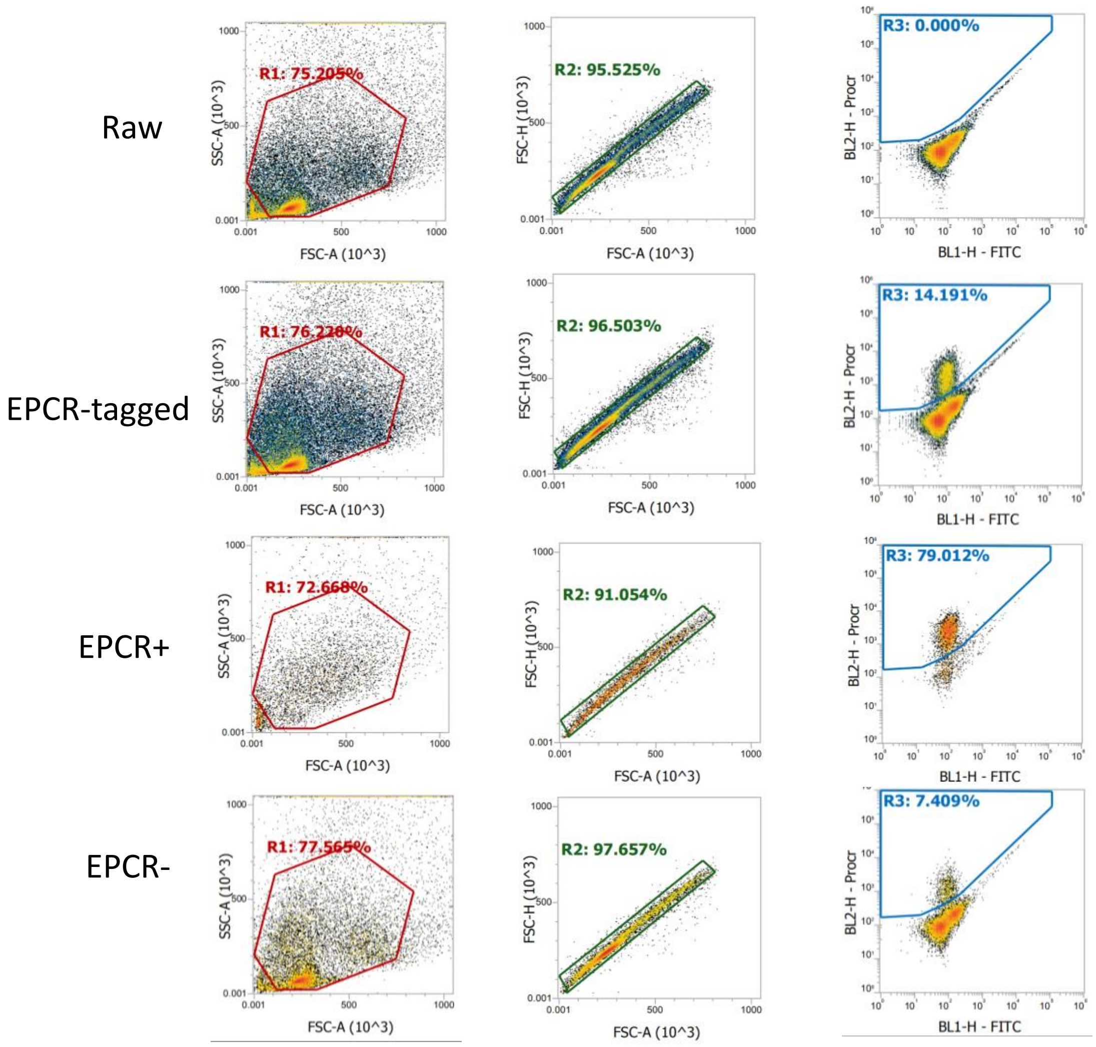

Supplementary Figure 5: Cell isolation by Magnetic Assisted Cell Sorting (MACS) procedure. Representative flow cytometry profile of cells isolated based on EPCR. Untagged (Raw), EPCR-tagged, EPCR+ and EPCR- cells were compared.

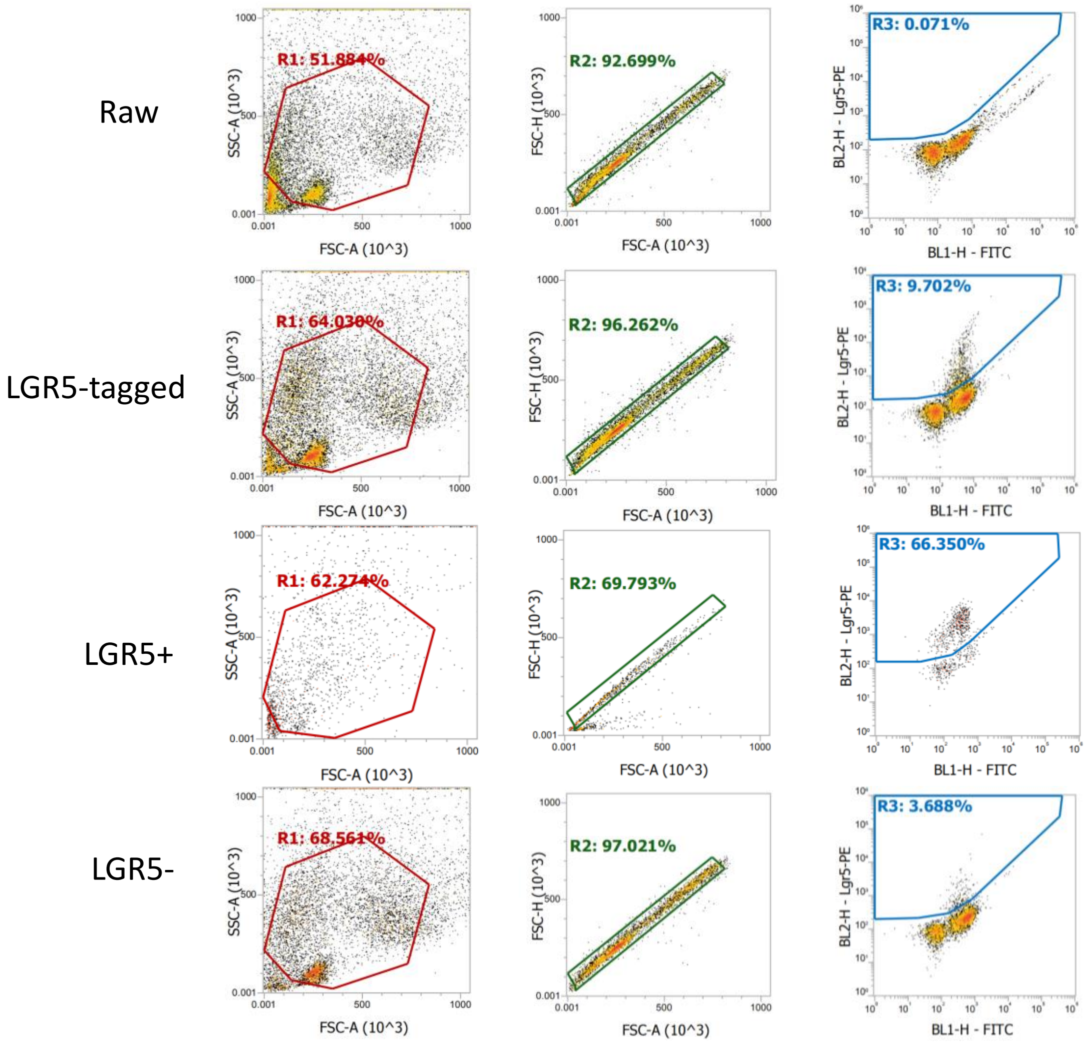

Supplementary Figure 6: Cell isolation by Magnetic Assisted Cell Sorting (MACS) procedure. Representative flow cytometry profile of cells isolated based on LGR5. Untagged (Raw), LGR5-tagged, LGR5+ and LGR5- cells were compared.
